# Osmolytes and *CsAQP* expression jointly influence water physiology in the peel and pulp of orange (*Citrus sinensis* (L.) Osbeck) fruit during postharvest water loss

**DOI:** 10.3389/fpls.2024.1475574

**Published:** 2024-10-21

**Authors:** Xiong Lin, Qingjiang Wei, Lingcai Zeng, Minxuan Zhan, Feng Li, Jinyin Chen, Qiaoli Ma

**Affiliations:** ^1^ Jiangxi Key Laboratory for Postharvest Technology and Nondestructive Testing of Fruits and Vegetables, Collaborative Innovation Center of Postharvest Key Technology and Quality Safety of Fruits and Vegetables, Jiangxi Agricultural University, Nanchang, China; ^2^ Citrus Science and Technology Backyard of Jinxian Country, Jiangxi Lufeng Ecological Agriculture Development Co., LTD, Nanchang, China

**Keywords:** postharvest orange fruit, water loss, water potential, aquaporins, *CsAQPs*, osmolytes

## Abstract

Water loss is a serious issue affecting the quality of postharvest horticultural products. Aquaporins (AQPs) regulate the transport of water across biological membranes, along the gradient of water potential, and may play a role in water loss. In this study, matured orange fruits (*Citrus sinensis*) stored at ambinent temperature (RH 85-95%) for 105 d showed that the weight loss persistently increased, and its rate peaked at 45–60 d and 90–105 d. Both water content and potential were higher in the pulp than in the peel. Water content rose before 60 d, and peel water potential fell with an increased gradient after 60 d. Comparing with peel, osmolytes such as soluble sugar, sucrose, glucose, fructose, and organic acids showed higher accumulation, and their levels were the lowest around 60 d. In contrast, soluble protein and inorganic minerals showed low levels of accumulation in the pulp. In total, 31 *CsAQP* genes were expressed in the fruit, and most of them were down-regulated in the peel but up-regulated in the pulp during storage. These genes were subsequently classified into four clusters based on their expression patterns. Genes in Cluster I — including *CsNIP1;1/2;1/2;2/2;3/3;1/4;1/6;1*, *CsTIP1;3/2;2/2;3/5;1/6;1*, *CsXIP1;1/1;2*, *CsSIP1;2*, and *CsPIP1;2* — were persistently up-regulated in the pulp for the 105 d of storage, especially at day 60, when some genes showed 103-fold higher expression. Pearson’s correlation and principal component analysis further revealed a significant positive correlation among weight loss rate, water content, and water potential gradient (R^2^ = 0.85). Indexes positively correlated with osmolyte content and Cluster I gene expression in pulp samples suggest that increased *CsAQP* gene expression in pulp is linked to faster water loss in oranges, particularly at 60 days postharvest.

## 1 Introduction

Water, a crucial component of living organisms, plays vital roles in plant growth, development, maturation, ripening, and senescence (Hou et al., 2019; H. et al., 2020; Lufu et al., 2020; Ha et al., 2021). Typically, water accounts for 70–90% of the fresh weight of most horticultural products (Canet, 1988). Notably, citrus fruits are among the most popular horticultural commodities in both Chinese and global markets (Lu et al., 2023). The peel of citrus fruits, which comprises the flavedo and albedo, contains essential oils, pigments, and cellulose, and its average water content is around 75% of its fresh weight (Czech et al., 2020). The pulp — the edible portion of citrus fruits — consists of succulent segments filled with juice sacs and has a water content that is nearly 90% of its fresh weight (Lu et al., 2023). During postharvest storage, fresh horticultural products are highly prone to water loss through continuous transpiration. This loss, which often exceeds 90% of the total weight loss, is frequently synonymous with mass loss (Lufu et al., 2020). Excessive water loss (e.g., exceeding 5% of the initial weight of orange fruit) can inflict irreversible damage on cellular physiological and metabolic activities. This damage manifests as wilting; shriveling; and the loss of glossiness, firmness, texture, and flavor, rendering the fruit more susceptible to abiotic and biotic stresses. This ultimately leads to senescence and renders the fruit unmarketable (Grierson and Wardowski, 1978; Ben-Yehoshua and Rodov, 2002; Paniagua et al., 2013; Cronjé et al., 2017; Wei et al., 2019).

The physiological and biochemical characteristics of plant cells, such as their water potential and the levels of osmotic solutes, play a pivotal role in regulating water loss (Landrigan et al., 1996; Hou et al., 2019; Ghosh et al., 2021). Water potential, which is positively correlated with water content, acts as a direct driver of water flow (Lockley et al., 2021). Previous studies have demonstrated that the water potential of citrus peel decreases during storage, leading to increased water loss in fruits. Severe dehydration and abrupt changes in water potential can also induce physiological impairments in the peel (Cronjé et al., 2017). Similarly, research on apples with watercore revealed a steep water potential gradient from the normal outer parenchyma to the watercore region, which led to sustained water transport toward the watercore region (Wada et al., 2021). Under conditions of low water potential, plant cells maintain osmotic pressure by increasing the levels of osmotic solutes, including amino acids, soluble sugars, proteins, inorganic ions (such as K^+^, Ca^2+^, and Mg^2+^), and other osmoprotectants like proline and betaine. This adaptive response enhances water absorption and can also reduce water loss (Ozturk et al., 2021; Wang et al., 2022). Postharvest fruits experience water shortage stress because they cannot receive water from the parent tree. However, little research has been conducted to investigate the changes in water potential and osmolytes in both the peel and pulp of postharvest citrus fruit, as well as their relationship with water loss.

Aquaporins (AQPs) are membrane channels belonging to the major intrinsic proteins (MIPs) superfamily. AQPs facilitate the transport of water, hydrogen peroxide, small neutral solutes, and gas molecules across biological membranes (Groszmann et al., 2017; Luang and Hrmova, 2017). In plants, AQPs play a pivotal role in short-distance transmembrane water transport, and plants can modify their water status by regulating the number or activity of AQPs under water stress and other abiotic stress conditions (Kapilan et al., 2018; Singh et al., 2020). AQPs are categorized into five major subfamilies based on the sequence similarity of their coding genes and their subcellular localization: plasma membrane intrinsic proteins (PIPs), tonoplast intrinsic proteins (TIPs), nodulin26-like intrinsic proteins (NIPs), small basic intrinsic proteins (SIPs), and uncategorized intrinsic proteins (XIPs) (Singh et al., 2020). While PIPs, TIPs, NIPs, and SIPs are commonly found in major higher plant species, XIPs are absent in monocots and the Brassicaceae family (Maurel et al., 2015). Previous research identified 34 AQP genes in the genome of sweet orange, including *11 PIPs*, nine *TIPs*, eight *NIPs*, three *SIPs*, and three *XIPs* (Wei et al., 2019). Some of these genes were found to exhibit differential expression in the roots and leaves of two orange cultivars that had contrasting drought-tolerance activities. However, the expression patterns of these AQP genes during the ripening and storage of fruit were not explored.

In recent years, accumulating evidence from research on horticultural crops has highlighted the important roles of AQPs in fruit growth, development, and ripening especially under the water deficit condition. The changes of *AQPs* gene expression and activity under water stress will help plants to defense the dehydration by regulating water transport (Martins Cde et al., 2015; Wei et al., 2019). For instance, the ectopic expression of *PIP1;3* from the apple genome has been shown to increase fruit size and enhance drought tolerance in transgenic tomatoes (Wang et al., 2017). Meanwhile, the transcriptome analysis of pomegranate fruit revealed that the transcript abundance of *PgrPIPs* in this fruit was significantly higher than that of other subfamilies. Moreover, the mRNA levels of *PgrPIP1.3*, *PgrPIP2.8*, and *PgrSIP1.2* exhibited a significant linear relationship with water accumulation in the seed coat (edible part), which suggested that these genes may contribute to cell expansion in the outer seed coat (Liu et al., 2021). Similarly, papaya fruits with higher expression of TIPs and SIPs have been found to exhibit larger mesocarp cells and intercellular spaces (Burns et al., 2023). AQPs exhibit tissue- and cultivar-specific expression patterns, although their expression also depends on the stage of fruit development and ripening (Merlaen et al., 2018; Quirante-Moya et al., 2022). In a previous study, 20 AQP genes were found to show differential expression patterns between ‘Red Fuji’ and ‘Golden Delicious’ apples, which have contrasting ripening behaviors (Lv et al., 2023). During the fruit development stage, expression of *AQPs* genes in peel and pulp was inconsistent, and most of them were down-regulated toward ripening (Yunhao et al., 2019; He et al., 2022). However, some AQP genes have specifically been found to be up-regulated during ripening, such as *FaPIP1;1*, *FaNIP1;1*, and *FaPIP2;1* in strawberry fruit (Mut et al., 2008; Alleva et al., 2010; Molina-Hidalgo et al., 2015), *GjTIP* in *Gardenia jasminoides* (Gao and Guo, 2013), and *MaPIP2-10*, *MaPIP1-6*, *MaPIP2-7*, *MaSIP1-1* in banana (Hu et al., 2015), and *PaPIP1;4* in sweet cherry (Breia et al., 2020), This suggests the potential roles of these genes in enhancing fruit crispness and firmness and preventing cracking (Burns et al., 2023).

According to some studies, in the postharvest stage, the high expression of certain AQPs may be detrimental to water maintenance in fruits. For instance, increased water loss in postharvest tomatoes induced by exogenous melatonin was associated with the up-regulation of *SlPIP12Q*, *SlPIPQ*, *SlPIP21Q*, and *SlPIP22* (Sun et al., 2015). A similar phenomenon was observed in pears, where accelerated water loss was linked to the increased degradation of cell wall polysaccharides and *AQP* gene expression (Ma et al., 2023). Notably, the overexpression of the *CsNIP5;1* gene in citrus fruit and callus tissues was found to inhibit other *AQP* genes (such as *CsPIP1;1*, *CsPIP2;4*, and *CsTIP1;2*) and alleviate water loss (Zhang et al., 2021). Nevertheless, given the large number of AQPs present in plant cells, their tissue-specific expression, and their functional redundancy, the regulatory mechanisms of AQPs in the context of postharvest water loss in fruits remain poorly understood.

To explore the key factors affecting postharvest water loss in citrus fruits, we focused on the physiological differences in water status between the peel and pulp of Newhall navel orange (*Citrus sinensis* (L.) Osbeck) fruits during a 105-day storage. The differences of water physiology indexes such as the water potential, water content, weight loss rate and osmolyte accumulation, and the spatial and temporal expression levels of all *CsAQP* genes were examined regularly. additionally, the relationship between *CsAQP* expression and water loss was analyzed in detail. Overall, the results of this study will provide a clue for controlling postharvest water loss of citrus fruits.

## 2 Materials and methods

### 2.1 Fruit materials

About 600 kg mature Newhall navel orange fruits were harvested from a local commercial orchard in Ganzhou City, Jiangxi Province, China, and were soon transported to the laboratory within six hours. After removing field heat in a ventilated environment for two days, the fruits with uniform size and free from any visible injury were manually cleaned, air dried, and singly bagged with a polyethylene bag (18 cm × 14 cm × 5 μm), then stored at normal temperature (8 to 12°C, RH 85 to 95%) for about three months. Before this experiment, we have conducted an 84-day storage experiment in 2017.

Samples of peel and pulp were randomly collected from 20 fruits at 0, 15, 30, 45, 60, 75, 90 and 105 d during storage, and immediately cooled with liquid nitrogen, then stored in the refrigerator at -80°C for detecting osmolytes accumulation and *CsAQPs* gene expression.

### 2.2 Weight loss estimation

Sixty fruits were randomly selected and averagely divided into three groups for weight loss estimation. Fruit weight was individually recorded at each sampling time points. The accumulated weight loss was calculated by the change from initial weight (at 0 d) to the final weight (at each time point). And the average weight loss was calculated by the weight loss within each 15 days interval. The calculation formula is as follows:

Accumulated weight loss (%) = (W_0_−W_n_)/W_0_×100%

Average weight loss (%) = (W_n_−W_n+15_)/W_n_×100%

Note: The ‘W’ and ‘n’ indicate fruit weight and days of storage, respectively.

### 2.3 Water content estimation

To detect the water content in peel and pulp, 10 mm wide peel from the equatorial region were sliced and two segments on the opposite position were collect. Six to seven fruits for each biological group and totally 20 fruits were used. The peel and pulp samples were weighted before and after drying at 80°C for 96 h. The water content was calculated according to: (fresh weight - dry weight)/(fresh weight), and was expressed as %.

### 2.4 Water potential measurement

The water potential was measured using a PSYPRO water potential datalogger equipped with C-52 sample chamber and L51 probe (Wescor, South Logan, UT, USA). As described previously (Alférez et al., 2003), 10-mm disks of the fruit peel were excised from the central outer region of the fruit (region with the highest circumference) using a cork borer. These disks were then immediately placed in a sample chamber to test the water potential of the peel. The juice sacs of orange fruit are fragile, and the pulp water potential cannot be estimated directly. Hence, the juice sacs were squeezed to obtain fruit juice. A filter paper fully soaked with the juice was placed in the sample chamber to test the water potential of the pulp. The PSYPRO water potential system was previously calibrated with NaCl solutions of known concentrations. To ensure initial water vapor equilibrium, water potential measurements were done at least 30 minutes after setting the sample into the chamber. For each water potential measurement, nine fruits were used.

### 2.5 Osmolytes measurement

#### 2.5.1 Soluble sugar

Soluble sugar concentration was measured by the anthrone colorimetric method. Sample extraction and testing procedures are performed according to our published paper (Ma et al., 2021). One grams of flesh tissue was homogenized using Retsch-MM 400 mixer mill apparatus (Retsch Inc.; Newtown Pa., Germany), then mixed with 100 mL distilled water and bathed in boiling water for 30 min. After centrifugation (Hettichi-UNIVERSAL 320R, Germany) at 8000 × g for 10 min, 2 mL of 50 times diluted supernatant was reacted with 0.5 mL of 2% anthrone-ethyl acetate reagent and 5 mL of concentrated sulfuric acid on a boiling water bath for 60 s. The reaction solution was air cooled to room temperature and was then detected the absorbance at 630 nm by an ultraviolet-visible (UV–vis) spectrophotometer (UV-2600, Shimadzu, Kyoto, Japan). The result was ultimately expressed as mg/g of fresh weight. The experiment was biologically repeated at least three times.

#### 2.5.2 Soluble protein

Soluble protein concentration was assayed by Coomassie brilliant blue G-250 binding method. Two grams flesh tissue was homogenized using Retsch-MM 400 mixer mill apparatus (Retsch Inc.; Newtown Pa.,Germany), then mixed with 5.0 mL phosphate buffer. After centrifugation (Hettichi-UNIVERSAL 320R, Germany) at 12000 × *g* for 20 min at 4°C, 1.0 mL of supernatant was reacted with 5.0 mL Coomassie brilliant blue G-250 solution for 2 min. The absorbance value of reaction solution was detected at 595 nm by an ultraviolet-visible (UV-Vis) spectrophotometer (UV-2600, Shimadzu, Kyoto, Japan). Soluble protein concentration was calculated the by a prepared standard curve, and the result was expressed as mg·g^-1^ fresh weight. The experiment was biologically repeated at least three times.

#### 2.5.3 Components of sugars and organic acids

Referring to our previous methods (Yang et al., 2022) for sugar and organic acid detection, 4 g freezed powder sample was water bathed with 25 mL of 80% ethanol at 35°C for 20 min, then centrifuged at 10,000 *g* for 15 min. One milliliter of supernatant was evaporated at 30°C until gelatinous using a rotary evaporator (5305, Eppendorf AG 122,339 Hamburg, Germany), then re-dissolved in 1 mL of ultrapure water. After filtered through a 0.45 μm or 0.22 μm film, the sugars or acids in the solution were quantified by using HPLC (LC-20A, Shimadzu, Japan) equipped with RID detector or diode array detector. The sample volume was 20 μL. The chromatographic condition for sugar detection was: Waters NH_2_ column (4.6 mm × 250 mm, 5.0 μm), column temperature kept at 35°C; mobile phase, acetomitrile: water (85: 15) at a rate of 0.8 mL·min^-1^. The chromatographic condition for organic acid detection was: Waters C18 column (4.6 mm × 250 mm, 5.0 μm), column temperature kept at 25°C; mobile phase, 0.01 mol·L^-1^ H_2_SO_4_ (using KOH to adjust the *p*H to 2.6) at a rate of 0.5 mL·min^-1^. The concentration of sugar and acid in the sample was determined based on the standard curve derived from the sugar and acid standard, and was ultimately expressed as mg/g of fresh weight. The experiment was biologically repeated at least three times.

#### 2.5.4 Mineral elements

About 0.5 g dry sample was weighed in a 25 mL ceramic crucible, and baked in a muffle furnace at 550°C for 5 h. The ash was taken out slightly and cooled to room temperature, then mixed with 10 mL of acid solution (60% water, 30% hydrochloric acid, 10% nitric acid), 30 min later, the filtrate was directly detected by ICP-OES5110 (Agilent, America) ICP-AES. The calculation formula is: fresh weight concentration of mineral elements = dry weight concentration of elements/(100% - tissue water content %). The result was ultimately expressed as mg/g of fresh weight. The experiment was biologically repeated with at least three times.

### 2.6 RNA extraction, cDNA synthesis and qRT-PCR

About 2 g of peel and pulp tissues were used to extract the Total RNA by TRZOL reagent according to the procedure of previous report (Yang et al., 2022) and three biological replicaes were performed. One microgram (μg) of high-quality total RNA was used for first-strand cDNA synthesis by using a Prime Script RT Reagent kit with a gDNA Eraser (TaKaRa, Dalian-China). qRT-PCR was performed in a 10 μL reaction volume by using SYBR Premix Ex Taq II kit (TaKaRa, Dalian, China) according to the manufacturer’s protocol. Primer sequence were synthesized in accordance with our published paper (Wei et al., 2019). The reaction started with an initial incubation at 95°C for 30 s, followed by 40 cycles of 95°C for 5 s, and 60°C for 30 s. Expression of gene was calculated using 2^− ΔΔCt^ method. qRT-PCR was conducted with three biological replicates, and each biological replicate had three technical replicates.

### 2.7 Statistical analysis

ANOVA analyses, Pearson’s correlation analysis and principal component analysis (PCA) were performed with SPSS Statistics 20.0 software (IBM SPSS Inc., Chicago, IL, USA) at the level of *P* < 0.05, or 0.01. Plots were graphed by GraphPad Prism 8.0 software (GraphPad, San Diego CA, USA). Gene expression cluster plot were conducted by TBtools (Chen et al., 2023). All data are expressed as the mean ± standard deviation (SD).

## 3 Results

### 3.1 Changes in weight loss, water content, and water potential

**Figure 1:**
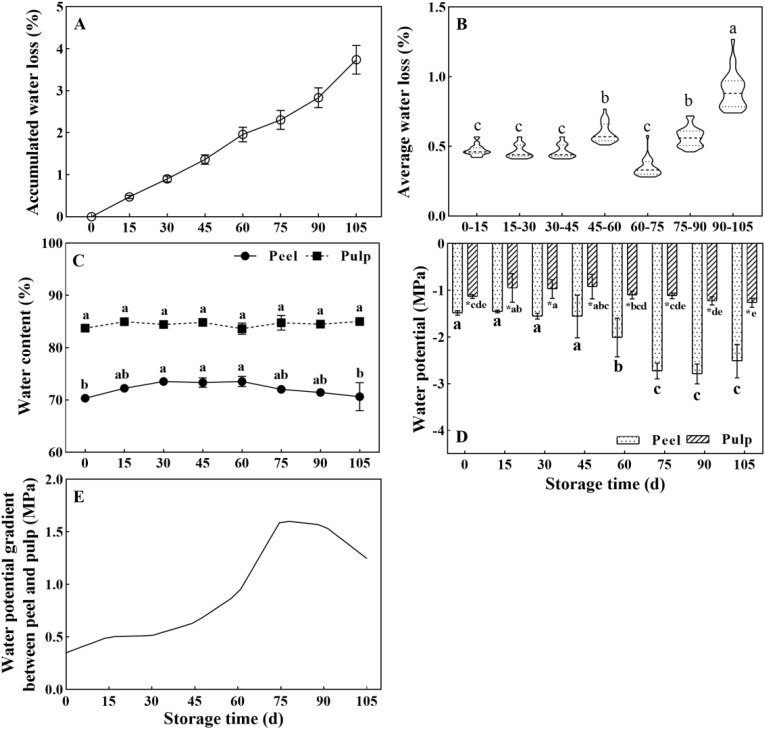
Changes in water-related parameters in Newhall navel orange fruit during storage. **(A)** Cumulative weight loss rate and **(B)** average weight loss rate in the whole fresh fruit per 15 days of postharvest storage; **(C)** Water content and **(D)** water potential in the peel and pulp; **(E)** Curve showing changes in the water potential gradient between the peel and pulp during storage. The values were presented as means ± SD (n ≥ 3). Different lowercase letters mean significant differences in the same tissue at different storage times, asterisk means significant difference between peel and pulp, *P* < 0.05 (The same as below).

The water loss persistently increased during storage and reached 3.74% at the end of the storage period (105 d) (**Figure 1A**). However, the water loss rate did not increase in a uniform manner (**Figure 1B**). The average weight loss rate per 15 days was about 0.45% between 0 and 45 d and 60 and 90 d, but it was 0.60% and 0.90% during 45–60 d and 90–105 d of storage, respectively. This indicated an acceleration of water loss during the later phase of storage. The mean water contents were 70.36% and 83.76% in the peel and pulp at the beginning of storage, respectively. The water content of the pulp did not change significantly in the pulp. However, the water content of the peel increased between 0 and 60 d and decreased thereafter (P < 0.05) (**Figure 1C**). A similar phenomenon was also observed in samples from 2017 after an 84-day storage duration (**Supplementary Figure 1**).

Consistent with the difference in the water content between the pulp and peel, a water potential gradient was detected between the pulp and peel. Notably, the water potential in the pulp was significantly higher than that in the peel during storage (**Figure 1D**). The water potential in the peel showed no change between 0 and 45 d of storage (about -1.48 MPa) but dropped from 60 d onwards, reaching -2.79 MPa at 90 d. Meanwhile, the water potential in the pulp showed limited changes. It increased from -1.13 MPa (0 d) to -0.92 MPa (45 d) and was then maintained at -1.1 to -1.26 MPa between 60 and 105 d. The water potential gap between the peel and pulp was small and slowly increased before the 60-d time point. However, it increased drastically between 60 and 90 d, and the highest increase was observed at 75 d (**Figure 1E**). Overall, the data suggested that the later storage period (after 60 d) is the key period for changes in water physiology as well as water loss in orange fruit.

### 3.2 Changes of organic osmolytes

**Figure 2:**
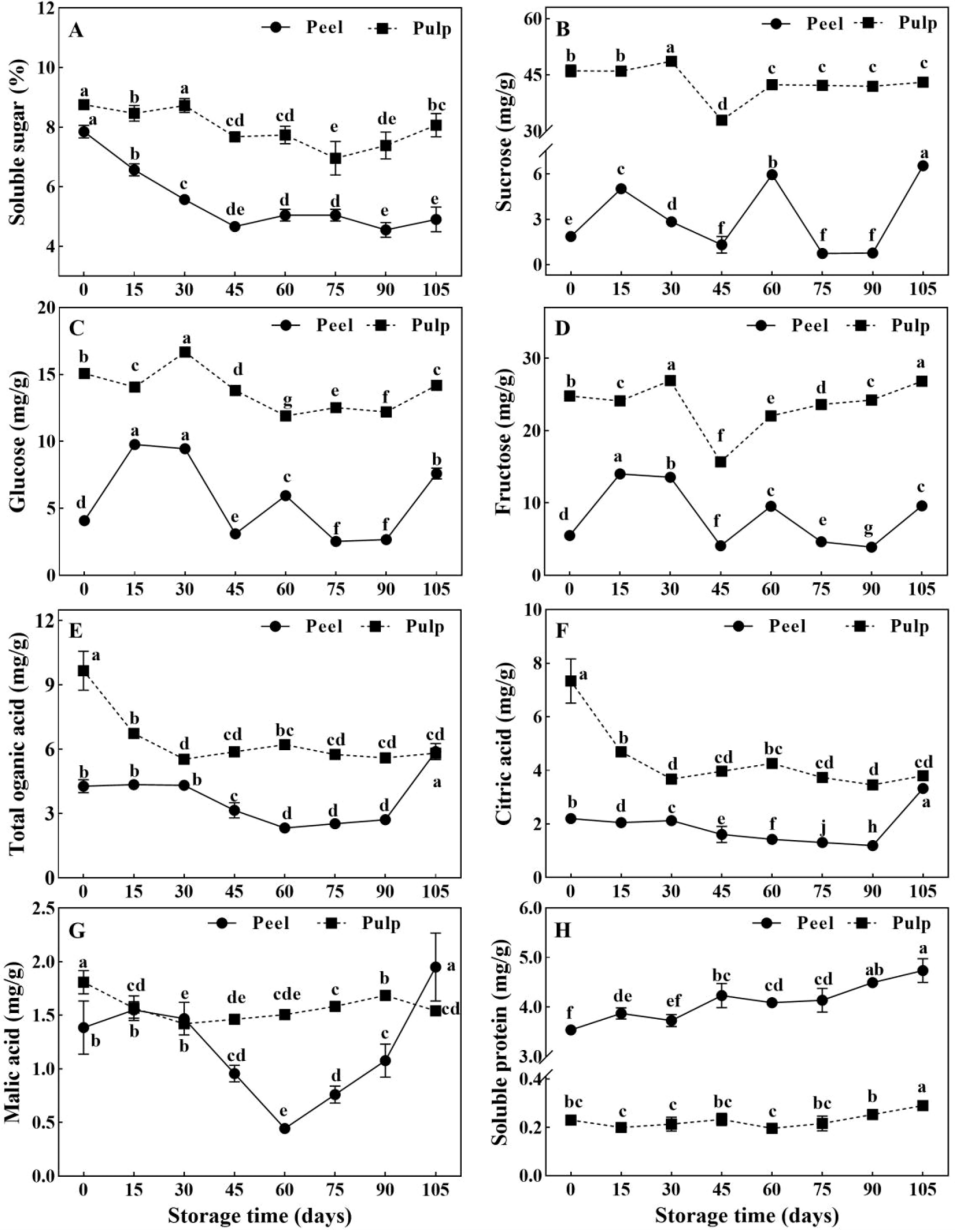
Changes of organic osmolytes in Newhall navel orange fruit during storage. **(A)** Soluble sugar, **(B)** sucrose, **(C)** glucose, **(D)** fructose, **(E)** organic acid, **(F)** citric acid, **(G)** malic acid and **(H)** soluble protein in peel and pulp of Newhall navel orange during storage.The values were presented as means ± SD (n = 3). Different lowercase letters mean significant differences in the same tissue among different storage times with *P* < 0.05.

Soluble sugar, organic acids, and soluble protein are important organic osmolytes in plant cells, and their levels can affect the water potential. The concentration of soluble sugars (**Figure 2A**), sucrose (**Figure 2B**), glucose (**Figure 2C**), fructose (**Figure 2D**), total organic acids (**Figure 2E**), and citric acid (**Figure 2F**) was significantly higher in the pulp than in the peel. Meanwhile, the soluble protein concentration (**Figure 2H**) was 15-fold higher in the peel. Sucrose, glucose, fructose, and soluble sugar in pulp increased and peaked at 30 d, then decreased to valley around 60 d (**Figures 2A–D**). In the peel, soluble sugar levels decreased rapidly before 45 d but remained relatively stable thereafter (**Figure 2A**). However, the sucrose, glucose, and fructose concentrations showed the same trend of variation throughout the storage period, exhibiting small peaks at 60 d (**Figures 2B–D**). The levels of citric acid and malic acid, the main organic acids in citrus fruits, sharply declined in the pulp before the 30-d point and remained low thereafter. Meanwhile, the levels of these acids in the peel did not show any change before 30 d, although they showed a subsequent decrease until 60 d (**Figures 2E, F**). Soluble protein concentration continuously increased in the peel from to 3.54 ± 0.10 mg·g^-1^ to 4.74 ± 0.24 mg·g^-1^, but a tiny change around 0.23 ± 0.03 mg·g^-1^ in the pulp during storage.

### 3.3 Changes of inorganic osmolytes

**Figure 3:**
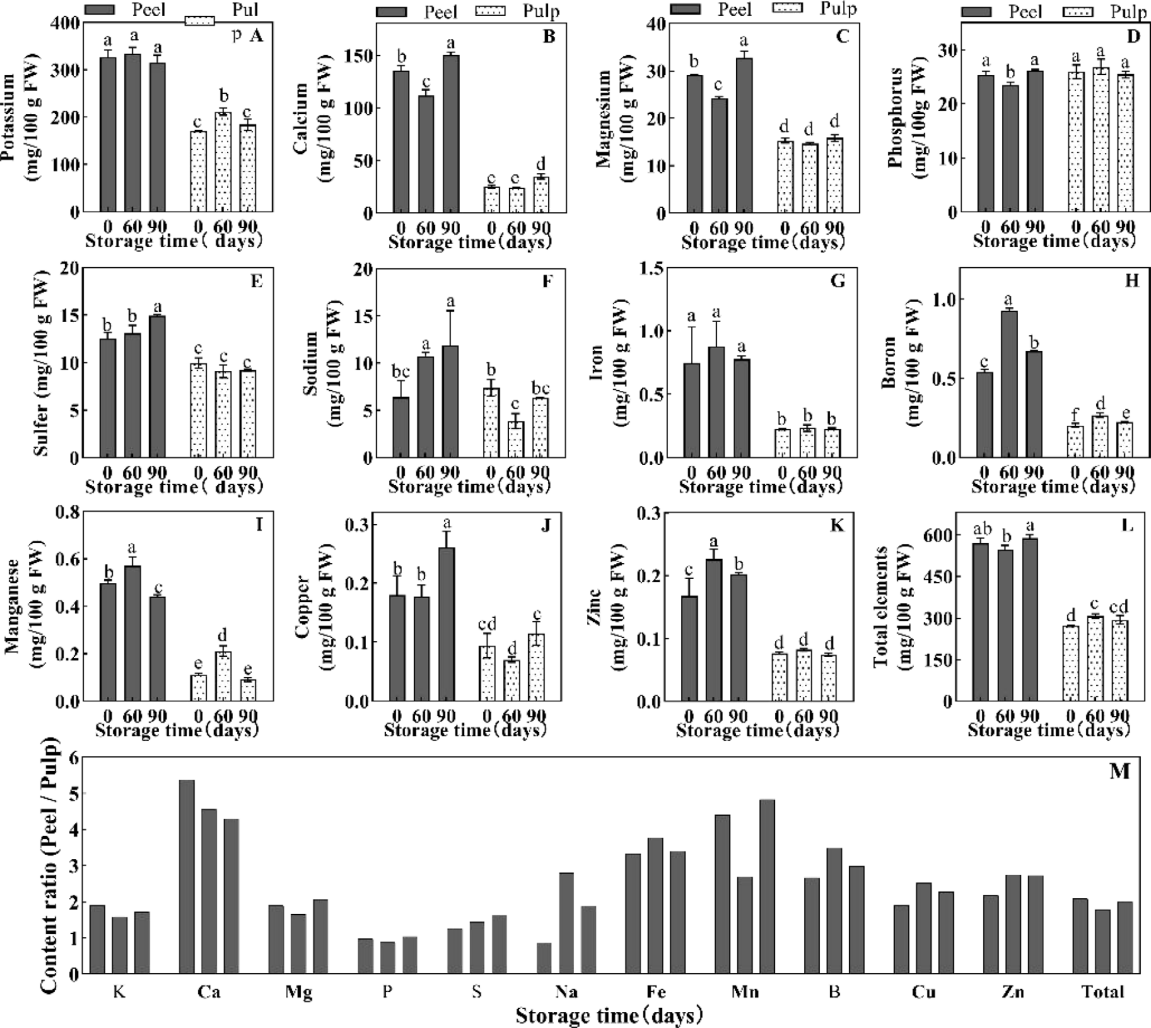
Element concentration of **(A)** K, **(B)** Ca, **(C)** Mg, **(D)** P, **(E)** S and **(F)** Na, **(G)** Fe, **(H)** B, **(I)** Mn, **(J)** Cu and **(K)** Zn, and the total element concentration **(L)** in the peel and pulp of Newhall navel orange at 0 d, 60 d and 90 d post storage, and **(M)** the element concentration ratio of peel/pulp at the same storage period. The values were present as means ± SD (n=3). Different lowercase letters mean significant differences in the same tissue among different storage times with *P* < 0.05.

Many minerals are involved in the regulation of water balance and osmotic homeostasis in plant cells. Herein, we determined the concentrations of various mineral elements in the peel and pulp at 0, 60, and 90 d of postharvest storage. Considering that water is the main component of plant tissue, the change of fresh weight concentration of solute can better reflect the change of tissue water potential. In this experiment, the element dry weight concentration data (**Supplementary Figure 2**) was converted into fresh weight concentration (**Figure 3**). All minerals other than phosphorus were found to show significantly higher levels in fresh peel samples than in fresh pulp samples (**Figures 3A–L**). Potassium and calcium were the most abundant minerals in orange fruit, and their levels in the peel were about 2- and 5-fold higher than their levels in the pulp (308–353 vs 158–194 mg·100 g FW-1 for potassium and 118–147 vs 22–34 mg·100 g FW-1 for calcium) (**Figures 3A, B, M**). Meanwhile, magnesium, Potassium, sulfur, and sodium showed moderate accumulation in the fruit (3.85–29.19 mg·100 g FW-1), and their concentrations were 1–2-fold higher in the peel than in the pulp (**Figures 3C–F, M**). Iron, boron, manganese, copper, and zinc (less than 1 mg·100 g FW-1) were present in much smaller amounts in orange fruit, but their accumulation in the peel was significantly higher than their accumulation in the pulp (**Figures 3G–K**). For example, iron, boron, and manganese levels were 3–4 times higher in the peel than in the pulp, while copper and zinc levels were 2–3 times higher (**Figure 3M**). This difference in the levels of mineral elements indicated the presence of a huge concentration gradient between the peel and the pulp, which may aggravate water and weight loss in fruit during storage. During storage, the potassium content did not change in the peel but showed a 23% increase in the pulp at 60 d (**Figure 3A**). The concentrations of calcium, magnesium, and phosphorus in the peel decreased at 60 d and recovered at 90 d; however, the concentrations of these elements, except calcium, did not show any change in the pulp at 90 d (**Figures 3B–D**). The concentrations of other elements in the peel increased at 60 d and/or 90 d, but showed only slight changes in the pulp (**Figures 3E–K**). The absolute increase in the concentrations of all detected elements during storage was negligible relative to their total concentrations (**Figure 3L**).

### 3.4 Relative expression of CsAQPs in the peel and pulp

**Figure 4:**
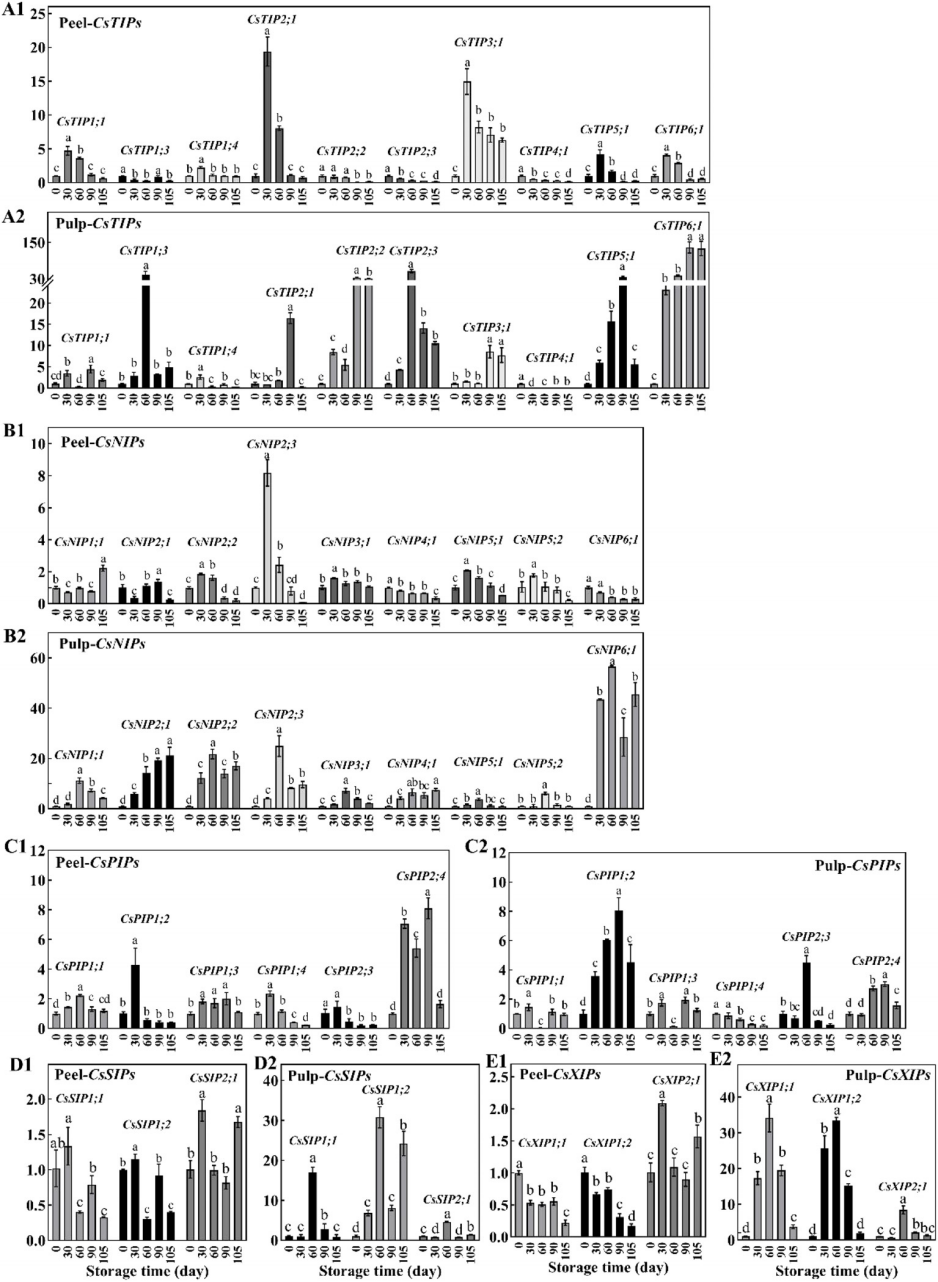
The relative expression of *CsAQPs* in peel (1) and pulp (2) of Newhall navel orange at 30, 60, 90, and 105 d comparing to 0 d, including **(A)** ten *CsTIPs*, **(B)** nine *CsNIPs*, **(C)** six *CsPIPs*, **(D)** three *CsSIPs* and **(E)** three *CsXIPs*. Different lowercase letters mean significant differences in the same tissue among different storage times with *P* < 0.05.

AQPs are proteins that mainly act as water channels and facilitate the passive transport of water. There are 34 *CsAQP* genes in the sweet orange genome. Of these, 31 genes were found to show differential expression at different time point in the peel and/or pulp, including ten *CsTIPs*, nine *CsNIPs*, six *CsPIPs*, three *CsSIPs* and three *CsXIPs* (**Figure 4**). In the peel, the expression of most genes from the *CsTIPs* family showed 2–5-fold elevations during storage, peaking at 30 d. Notably, the expression of *CsTIP2;1* and *CsTIP3;1* was 10 times higher at 30 d vs 0 d. However, the expression of *CsTIP1;3*, *CsTIP2;2*, *CsTIP2;3*, and *CsTIP4;*1 in the peel showed a gradual decrease during storage (**Figure 4A1**). In the pulp, most *CsTIP* genes (except *CsTIP4;1*, which was down-regulated) were continuously up-regulated during storage (fold change, 2–133), with their expression peaking at 60 d or 90 d (**Figure 4A2**). Notably, *CsTIP1;3*, *CsTIP2;2*, *CsTIP2;3*, *CsTIP5;1*, and *CsTIP6;1* showed a 30-fold up-regulation.

The expression of nine *CsNIP* genes in the peel did not change during storage (fold change < 2, P value > 0.05), although *CsNIP2;3* expression increased at 30 d (**Figure 4B1**). In contrast, *CsNIPs* were abundantly expressed in the pulp. In particular, the expression of *CsNIP2;1*, C*sNIP2;2*, and *CsNIP6;1* increased by 20–60 fold during storage (**Figure 4B2**). Most members of the *CsPIPs* sub-family did not show significant changes in expression during storage, with the exception of *CsPIP2;4* in the peel and *CsPIP1;2* in the pulp. Additionally, *CsPIP2;4* and *CsPIP1;2* were up-regulated 4–8 fold in the peel (30-90 d) and pulp (60-90 d), respectively (**Figures 4C1, C2**). Three *CsPIPs* were significantly up-regulated by 2.86–30.77 fold in the pulp but did not show altered expression in the peel, except *CsPIP1;1* and *CsPIP1;2*, which showed reduced levels at 60 and 105 d, respectively (**Figures 4D1, D2**). The expression trends of *CsXIP1;1* and *CsXIP1;2* in the peel were opposite to those in the pulp. These genes were down-regulated at 105 d in the peel but were abundantly up-regulated at 30–90 d in the pulp. Meanwhile, *CsXIP2;1* was up-regulated at 60 d in both the peel and pulp (**Figures 4E1, E2**).

### 3.5 Classification of the 31 differentially expressed CsAQPs based on their expression trends during storage

**Figure 5:**
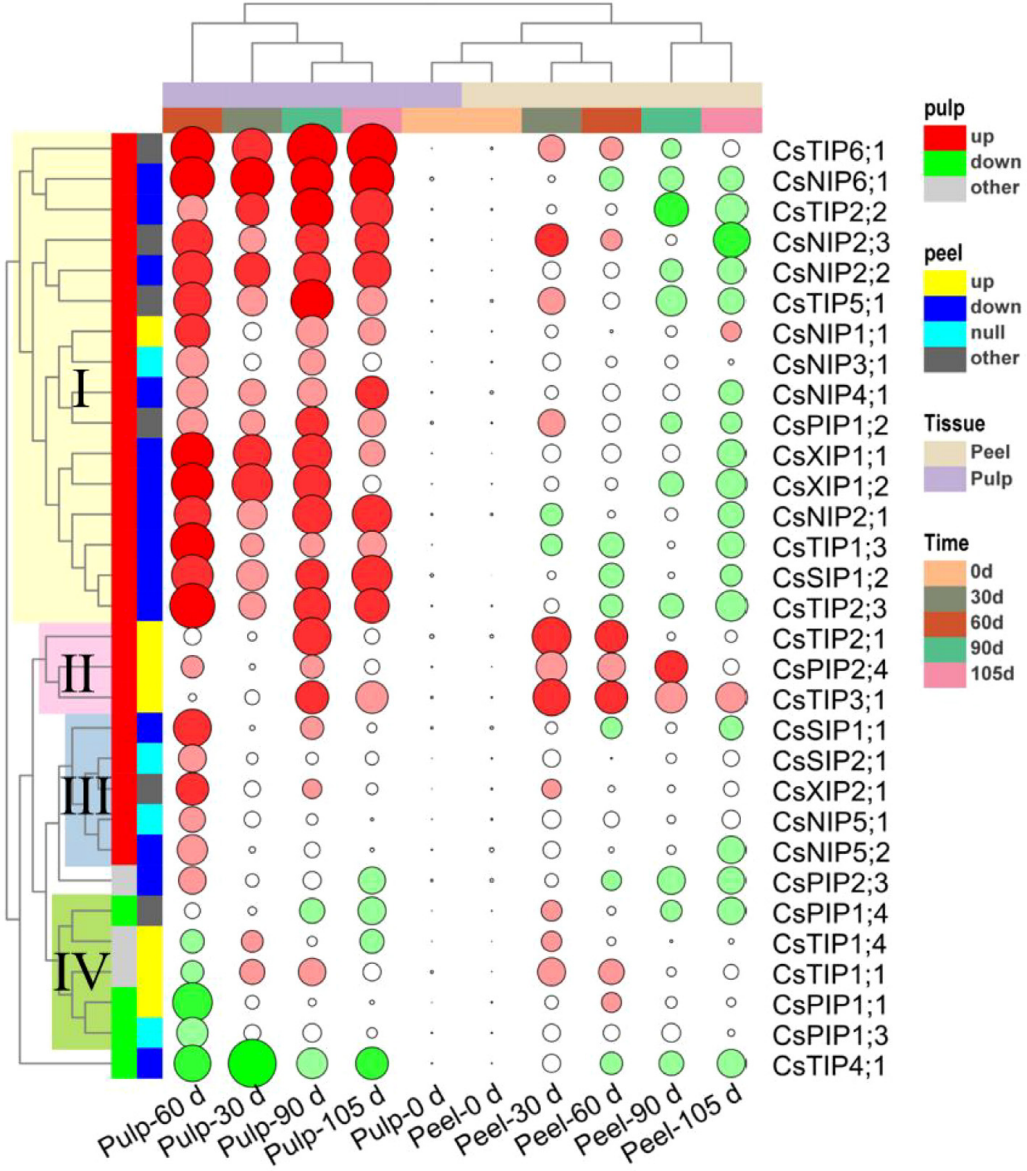
Clusters of the 31 differentially expressed *CsAQPs* genes based on their change mode in peel and pulp of Newhall navel orange during the 105 days storage. Green solid circle represents down-regulation, and red solid circle represents up-regulation (Fold change > 2 and *P* < 0.05), the lager circle area represents the greater fold change.

The abovementioned 31 differentially expressed *CsAQP* genes were clustered using a hierarchical clustering method based on the changes in their expression during storage; the results are displayed in **Figure 5**. More than 70% of the 31 *CsAQP* genes were down-regulated in the peel but up-regulated in the pulp (fold change > 2, and *P* < 0.05), and the peel samples were clustered independently from the pulp samples. The *CsAQP* genes showed distinct patterns of expression in the peel and pulp of Newhall navel oranges during postharvest storage, typically undergoing up-regulation in the pulp and down-regulation in the peel.

Based on time course analysis, the peel samples were clustered into two groups: 0–60 d and 90–105 d. During the 0–60 d period, 12 genes were up-regulated in the peel and 8 genes were down-regulated. Meanwhile, between 90 and 105 d of storage, 18 genes were up-regulated and 3 were down-regulated. These findings implied that the changes in *CsAQP* expression in the peel during storage can be classified into two different stages — the early stage (0–60 d), dominated by the gene up-regulation mode, and the later stage (90–105 d), dominated by the gene down-regulation mode. Meanwhile, 24 of the 31 *CsAQP* genes were up-regulated in the pulp over the entire storage period. The pulp samples could be clustered into three groups: 60 d, 30 d, and 90–105 d, with the 60 d group being clearly separated from the 30 d and 90–105 d groups. These results demonstrate the drastic up-regulation of *CsAQPs* in the pulp after 60 d of postharvest storage.

As shown in **Figure 5**, the *CsAQP* genes could be classified into four groups based on their relative changes in expression during storage. Cluster I contained 16 *CsAQP* genes, including four *CsNIPs* (*CsNIP1;1/2;1/2;2/2;3/3;1/4;1/6;1*), five *CsTIPs* (*CsTIP1;3/2;2/2;3/5;1/6;1*), two *CsXIPs* (*CsXIP1;1/1;2*), *CsSIP1;2*, and *CsPIP1;2*. These cluster I genes were markedly up-regulated in the pulp during storage but were significantly down-regulated in the peel between 90 and 105 d of storage. Genes in cluster II included *CsTIP2;1/3;1* and *CsPIP2;4* and were highly expressed in the peel throughout storage and were up-regulated in the pulp at 90 d of storage. Genes in cluster III, namely, *CsSIP1;1/2;1*, *CsXIP2;1*, and *CsNIP5;1/5;2*, were specifically up-regulated in the pulp at 60 d but did not show any altered expression in the peel. Finally, *CsPIP1;1/1;3*/*1;4* and *CsTIP1;1/1;*4 were grouped into Cluster IV and were down-regulated in the pulp, especially at 60 d, but were up-regulated in the peel between 30 and 60 d of storage. *CsTIP4;1* showed down-regulation in both the peel and pulp during storage. It was speculated that the *CsAQP* genes persistently expressed at high levels in the pulp while showing low expression levels in the peel may maintain active water flow from the pulp to the peel to keep the peel fresh during storage.

### 3.6 Overall analysis of the correlations among the water-related physiological indexes

**Figure 6:**
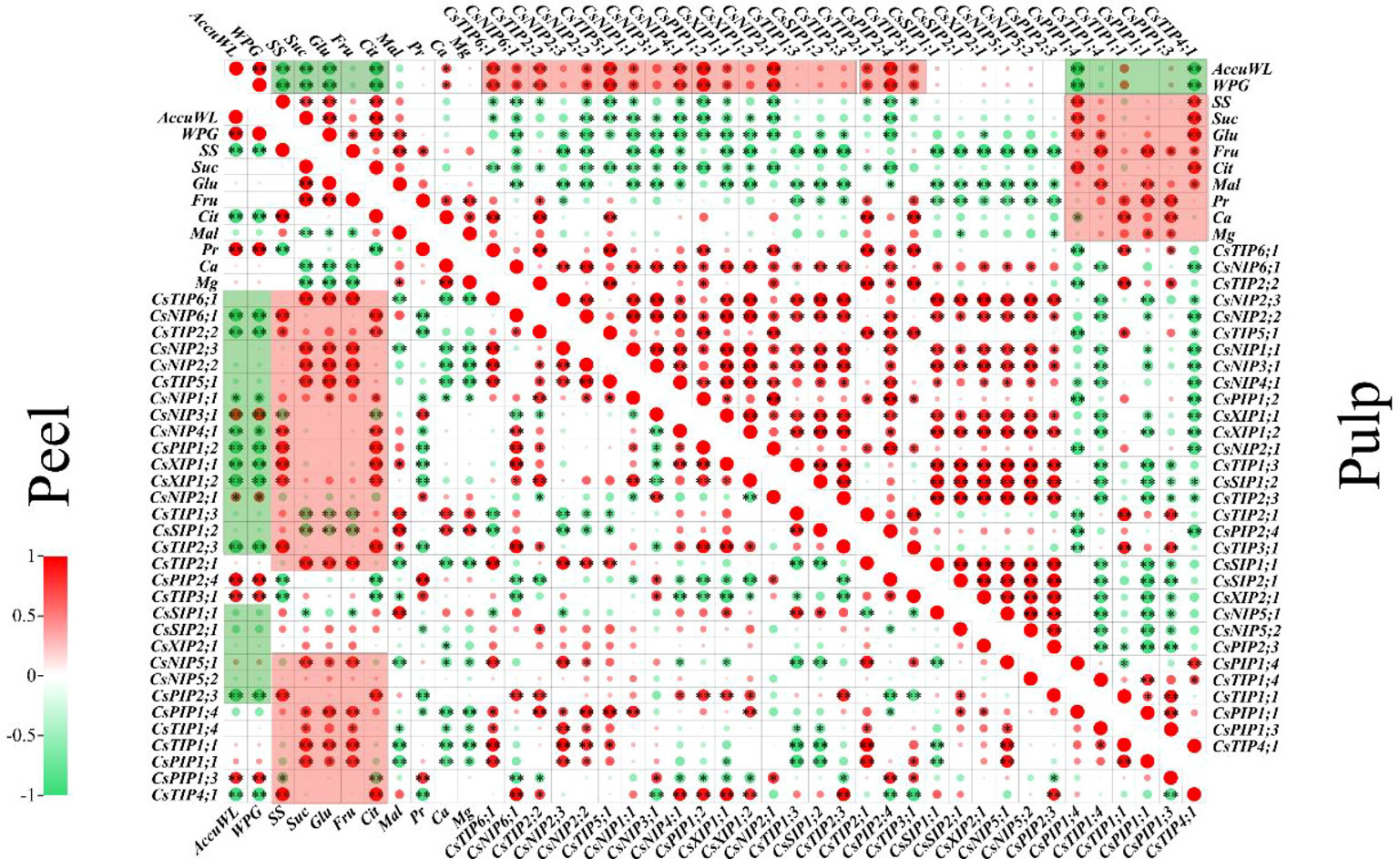
Pearson’s correlation matrix describes the association among weight loss, water potential, osmotic substances, and the *AQPs* gene expression in the peel or pulp samples at 0, 60, and 90 d. AccuWL, accumulation water loss; WPG, water potential gradient; SS, soluble Sugar; Suc, sucrose; Glu, glucose; Fru, fructose; Cit, citric acid; Mal, malic acid; Pr, protein. Single star ‘*’ and double stars ‘**’ represent the significance at the levels of *P* < 0.05 and 0.01, respectively. The red or green circle represents the positive or negative co-relationship with∣*R*∣> 0.8.

The variations in fruit water potential at 0, 60, and 90 days were markedly distinct. Therefore, data from these pivotal time points were chosen for this experiment to elucidate the correlation between water-related physiological indicators and the CsAQP gene. Pearson’s correlation coefficients for the relationships among weight loss, water potential gradient, sugar concentration, organic acid concentration, mineral concentration, and the expression of the 34 *CsAQPs* in the peel and pulp are displayed in **Figure 6**. The water potential gradient was positively correlated with the cumulative weight loss in both the peel and pulp, and both these indexes were negatively correlated with the soluble sugar and citric acid concentration (P < 0.05, ∣R∣ > 0.8). In the peel, weight loss and water potential gradient showed a strong positive correlation with the protein concentration and the expression of *CsNIP3;1*, *CsTIP3;1* and *CsPIP1;3*, but negatively correlated to *CsNIP6;1*, *CsTIP2;2/2;3*, *CsPIP1;2*, and *CsXIP1;1/1;2*. Meanwhile, the sugar, sucrose, glucose, fructose, and citric acid concentrations in the peel were positively correlated with the expression of most *CsAQP* genes, except *CsTIP1;3*, *CsSIP1;1/1;2*, and *CsPIP1;3*. Calcium and magnesium levels were positively correlated with each other and with *CsTIP1;3* and *CsSIP1;2* expression. In the pulp, the weight loss and water potential gradient showed a strong negative correlation with the concentration of sucrose and fructose and expression of *CsPIP1;4* and *CsTIP4;1*. However, these indexes were positively correlated with the expression of *CsTIP2;2/5;1/6;1*, *CsNIP2;1/4;1*, and *CsPIP1;2*. Osmolytes (soluble sugar, sucrose, glucose, fructose, citric acid, malic, protein, calcium, and magnesium) showed a negative correlation with the *CsAQP* genes in clusters I, II, and III and a positive correlation with the genes in cluster IV.

### 3.7 Principal component analysis of global changes in the peel and pulp during 0–90 d of postharvest storage

**Figure 7:**
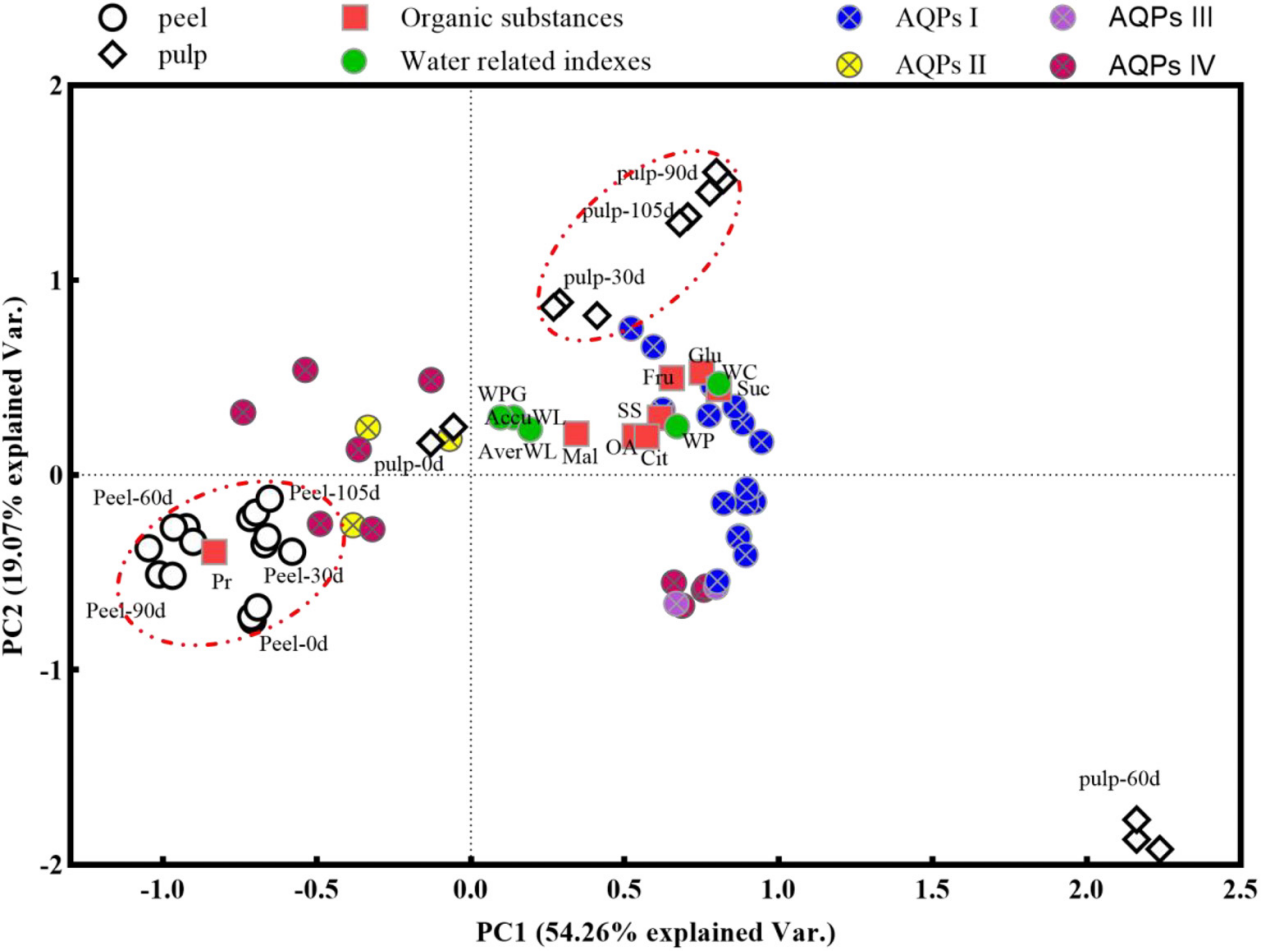
Principal component analysis (PCA) plot the global changes of water loss-related indexes using the data from peel and pulp samples during storage. Gene names of *CsAQPs* I, II, III, and IV have been showed in **Figure 4**. WC, water content; AverWL, average water loss; WP, water potential; WPG, water potential gradient; AccuWL, accumulation water loss; SS, soluble Sugar; Fru, fructose; Glu, glucose; Suc, sucrose; OA, organic acid; Cit, citric acid; Mal, malic acid; Pr, protein.

PCA was carried out based on data obtained from the peel and pulp samples after 0, 30, 60, 90, and 105 d of postharvest to further explore the changes in water-related physiological indexes (water content, water loss, and water potential) with storage time (**Figure 7**). Peel samples showed an obvious separation from pulp samples (except pulp-0d) on opposite directions of principal component 1 (PC1), which explained 54.26% of the total variance between samples. Meanwhile, the pulp-30d, -90d, and -105d samples were separated from all peel samples as well as pulp-60d samples on PC2, which explained 19.07% of the total variance. This suggested that the overall variation was primarily due to differences between pulp and peel tissues, while storage time was a secondary contributor to these variations. The peel samples showed close clustering on both PC1 and PC2. Additionally, they showed a significant positive correlation with the mineral content (brown triangles), protein concentration (a red square), and expression of cluster II and IV *CsAQP* genes (yellow and pink circles) as well as a negative correlation with water-related indexes (green circles). For the pulp samples at 30, 90, and 105 d, water-related indexes (green circles), osmolytes (red squares), and cluster I and III *CsAQP* gene expression (blue and purple circles) were positively correlated with each other on PC1. This revealed that the protein concentration was the main factor influencing the water-related indexes in the peel, and the sugars and organic acids were the main factors influencing the water-related indexes in the pulp.

## 4 Discussion

Water is a major component of fruits and vegetables and directly influences their sensory quality, including their firmness, crispness, glossiness, flavor, stress resistance, and shelf life (Landrigan et al., 1996; Frenkel and Hartman, 2012; Eboibi and Uguru, 2018). In the model fruits tomato and strawberry, water accounts for as high as 92.5% (Hou et al., 2019) and 90.90% (Akhtar and Rab, 2015) for the fresh weight, respectively. Unlike these berry fruits, citrus fruits belong to the group of hesperidium fruits and have a thick leathery peel and succulent pulp. The water content in citrus fruits varies considerably among different parts of the fruit and also among different fruit varieties. The pulp of orange, mandarin, grapefruit, pummelo, and lemon — the most commonly cultivated citrus varieties in the world — contains 82.30–87.90%, 80.70–88.60%, 76.90–91.60%, 89.10%, and 88.98% water, respectively (Mbogo et al., 2010; Lu et al., 2023). Meanwhile, the water content of the peel — which is an inedible component of citrus fruits — varies between 68.1% and 80.4% and is lower than the water content of the pulp (Czech et al., 2020). In line with previous reports, in the present study, the water content in the peel and pulp of Newhall navel orange fruit was found to be around 83.76–85.03% and 70.36–73.54% during storage, respectively (**Figure 1C**). Moreover, the water content of the pulp did not change obviously during storage, while that of the peel increased during the first 60 d and decreased thereafter (*P* < 0.05) (**Figure 1C**). A similar phenomenon was observed in samples from the year 2017 within 84 d of storage (**Supplementary Figure 1**). It has been proposed that the decrease in water content may induce fruit ripening (Frenkel and Hartman, 2012). Harvested citrus fruit can be stored for as long as 2–6 months, and excess ripening during this prolonged storage is detected based on the deterioration of internal quality using indicators such as total soluble solids (TSS), titratable acidity (TA) and TSS/TA values. Research on the shelf life of orange fruit has indicated that the critical stage of postharvest quality decline occurs at approximately 60 days postharvest (Frenkel and Hartman, 2012; Ma et al., 2021), consistent with the trend of water content changes in the peel (**Figure 1C**). Consequently, we speculate that, in addition to sugar and acid levels, the peel water content could also serve as an indicator of quality and physiological stage in citrus fruit during postharvest storage. Similar methodologies have been applied for the prediction of strawberry fruit quality based on optical properties (Xie et al., 2021).

In contrast to growing fruits, which absorb water from the parent plant, harvested fruits face water deficiency and are highly susceptible to water loss during storage. The loss of 3–10% of the initial weight renders most fruits and vegetables unmarketable (Ben-Yehoshua and Rodov, 2002), and citrus fruits are no exception. Desiccation due to water loss results in weight loss and affects the appearance, firmness, and commercial value of fruits while also limiting their shelf life. Studies have demonstrated that, besides carbon loss through respiration, water loss attributable to transpiration and respiration accounts for over 95% of the total weight loss in fruits (Bovi et al., 2018; Lufu et al., 2019; Xanthopoulos, 2021). Hence, water loss is frequently used interchangeably with weight loss in research on postharvest fruits. In our study, weight loss was measured to monitor the water loss from navel oranges during storage. We found that the water loss increased with prolonged storage, but this increase was not uniform. Specifically, the rate of water loss was accelerated during the later storage period (**Figures 1A, B**), with the average weight loss rate per 15 days increasing from 0.45% at 0–30 d to 0.60% and 0.90% at 45–60 d and 90–105 d, respectively (**Figure 1C**). This indicated that water loss in harvested oranges was not only due to simple physical diffusion but actually represented a physiological water transmission process that was affected by intracellular factors, such as water potential, solute potential, and turgor (Hou et al., 2021).

The water potential within plant cells, as indicated by their water content (Lockley et al., 2021) is a critical driving force for water movement across cellular membranes. In line with previous findings (Czech et al., 2020), in this study, water content and potential of Newhall navel orange is consistently higher in pulp than that in peel during storage (**Figures 1C, D**), which leads to a sustain water potential gradient between the pulp and peel (**Figure 1E**). Studies on watercore apples have shown that a steeper water potential gradient, extending from the normal outer parenchyma to the watercore region, establishes a sustainable mechanism for water transport to the watercore (Wada et al., 2021). Similarly, the presence of a water potential gradient between the peel and pulp tissues of Newhall navel oranges may generate a continuous driving force for water movement from the pulp to the peel, ultimately resulting in water loss from the surface of the fruit. Furthermore, similar to the minimal changes in water content observed in the pulp, the water potential of the pulp remained relatively stable during storage, whereas the peel water potential demonstrated a sharp decline from 60 d onwards. Previous studies on ‘Marsh’ grapefruit and ‘Navelate Navel’ oranges have revealed a significant positive relationship between peel water potential decline and fruit water loss (Alferez et al., 2010; Cronjé et al., 2017). In our study, the notable increase in the water potential gap between the peel and pulp from 60 to 105 d (**Figure 1E**) may explain the accelerated rate of water loss observed after 60 d (**Figures 1A, B**). Moreover, correlation analyses conducted on both peel and pulp samples (**Figure 6**) further demonstrated the positive relationship between the water potential gradient and cumulative weight loss. Based on the above analysis, we can infer that the sharp changes in water potential in the peel (**Figure 1D**) influence the water dynamics in postharvest citrus fruits, determining the rate of water loss during storage.

Solute potential, a critical aspect of water potential in plant cells, is determined by the presence of osmolytes, such as soluble sugars, proteins, organic acids, and various inorganic ions (Ozturk et al., 2021; Wang et al., 2022). In this study, we observed that soluble sugars (sucrose, glucose, and fructose), organic acids (citric acid, malic acid, etc.), soluble protein, and mineral elements were significantly more abundant in the pulp than in the peel (**Figures 2A–H**, **3A–K**). Generally, the total concentration of organic and inorganic osmotic solutes is slightly higher in the pulp (averaging about 71.51 mg/g FW) than in the peel (averaging about 68.41 mg/g FW). Throughout the storage period, the concentration of sugars and acids decreased in the pulp, with notable declines occurring around 30–60 d (**Figures 2A–G**). This decrease likely led to an increase in the osmotic and water potential within the pulp. Correlation analysis revealed that soluble sugar and citric acid concentrations were negatively correlated with the water potential gradient and cumulative weight loss (**Figure 6**). However, the water content and potential in the pulp remained relatively stable during storage (**Figures 1C, D**). Sucrose and citric acid are the most abundant compounds in citrus pulp (Zhou et al., 2018). Sucrose metabolism plays a crucial role in determining the soluble sugar concentration and osmotic potential of ripening fruit (Balibrea et al., 2006). Additionally, during storage, the degradation of hexoses and organic acids via glycolysis and the tricarboxylic acid cycle (Luo et al., 2021) further adds water to the pulp. Therefore, the degradation of sugars and acids in pulp cells appears to be essential for maintaining the water potential and water balance in Newhall navel oranges during postharvest storage (**Figures 1C, D**). In contrast, in our study, the osmolytes in the peel tissues showed no notable changes, unlike the water potential. The water potential of the peel sharply declined from -1.48 MPa before 60 d to -2.79 MPa after 90 d, while only the amounts of soluble protein and mineral elements increased in the peel, although the sugar and acid concentrations fluctuated over time (**Figures 2A–G**, **3L**). These results align with previous observations demonstrating that the osmotic potential in citrus peels does not undergo significant changes unlike the water potential (Alferez et al., 2010). Similarly, in previous studies, fruits such as apricots, doughnut peaches, and nectarines treated with foliar sprays of Ca+B and Ca+Si did not show changes in osmotic potential despite increases in water potential and turgor potential (Quirante-Moya et al., 2022). In our study, soluble protein exhibited a positive correlation with the water potential gradient in the peel (**Figure 6**). Hence, its increase may contribute to a lowered water potential in the peel, enlarging the water potential gap between the peel and the pulp and accelerating water transmission from the pulp to the peel during storage.

AQPs, water channels belonging to a major superfamily of intrinsic proteins, facilitate water transport across cellular membranes along the water potential gradient (Kapilan et al., 2018). *AQP* genes are typically categorized into five major subfamilies: *PIPs*, *TIPs*, *NIPs*, *SIPs*, and *XIPs* (Singh et al., 2020). In the sweet orange genome, 34 AQP-encoding genes have been identified. Our investigation revealed that 91.17% (31 out of 34) of these *CsAQPs* were expressed in postharvest Newhall navel orange fruit, including ten *CsTIPs*, nine *CsNIPs*, six *CsPIPs*, three *CsSIPs*, and three *CsXIPs* (**Figure 4**). This proportion closely resembles the expression rate of *AQPs* in ripening *Lycium chinensis* (97.37% or 37 out of 38) (He et al., 2022) and developing bananas (85.11% or 40 out of 47) (Hu et al., 2015). However, it surpasses the expression rates observed in ripening apples (47.62% or 20 out of 42) (Lv et al., 2023) and developing watermelons (34.29% or 12 out of 35) (Lin et al., 2019). This variance may be due to the temporal, spatial, and cultivar-specific expression patterns of *AQPs*.

Numerous studies have highlighted the differential expression and activity of various *AQP* genes across various fruits, tissues, and developmental and ripening stages under water deficit that induced by unproper environmental conditions (Merlaen et al., 2018; Wei et al., 2019; Quirante-Moya et al., 2022). Environmental stresses such as drought and salinity stress can rapidly reduce the rate of water transport across the membrane, while plants control water flow inside and outside the cell by regulating the abundance and activity of aquaporins to maintain water balance (Kapilan et al., 2018). In citrus fruits, aquaporin expression may be influenced by several factors, including plant growth conditions, developmental stages, tissue types, and the duration and intensity of stress. An analysis of the transcription profile of *CsAQP* genes in the roots and leaves of drought-tolerant (HJ) and drought-sensitive (HH) cultivars under drought treatment revealed that most *CsPIPs* and *CsTIPs* were down-regulated in roots of both treated cultivars. In addition, the down-regulation of *CsPIP1;2*, *CsTIP3;2*, and *CsNIP2;1* in roots and up-regulation of *CsNIP1;1*, *CsNIP1;3*, *CsNIP4;1*, and *CsNIP5;1* in leaves revealed obvious differences between tolerant and sensitive cultivars during drought (Rodríguez-Gamir et al., 2011; Martins Cde et al., 2015). Most *CsAQPs* exhibited higher expression levels in the pulp than in the peel and showed a decreasing trend during fruit development and ripening processes. For instance, in sweet cherry, *AQP* genes were more highly expressed in the flesh than in the skin, with 16 out of 25 AQPs being down-regulated during later developmental stages (Yunhao et al., 2019). Similarly, during *Lycium barbarum* fruit ripening, 24 *AQPs* were down-regulated, while three were up-regulated (He et al., 2022). Studies on postharvest mandarin, sweet orange, and pummelo fruits have also revealed the down-regulation of many *AQPs* and the up-regulation of a few *AQPs* in the pulp (*NIP1;2*, *NIP5;1*, *PIP2;2*, and *TIP3;1*) (Ding et al., 2015). Consistent with observations in sweet cherry (Yunhao et al., 2019), *CsAQPs* exhibited higher expression in the pulp than in the peel in our study, with most (19 out of 27) being down-regulated in the peel (indicated by green circles in **Figure 5**). However, 24 out of 31 *CsAQPs* showed up-regulation in the pulp at one or more test stages (indicated by red circles in **Figure 5**). Particularly noteworthy was gene cluster I, which includes *CsTIP1;3*, *CsTIP2;2*, *CsTIP2;3*, *CsTIP5;1*, and *CsTIP6;1* in the TIP subfamily; *CsNIP2;1*, *CsNIP2;2*, and *CsNIP6;1* in the NIP subfamily; *CsSIP1;1, CsSIP1;2*, *CsXIP1;1*, and *CsXIP 1;2*. This gene cluster exhibited expression 10–130 times higher levels in the pulp at 60 d than at 0 d (see **Figures 4**, **5**). A study in pomegranate fruit showed that eight *PgrPIP* members exhibited relatively high levels of transcript accumulation in both the inner and outer seed coats, with the transcription levels of *PgrPIP1.3*, *PgrPIP2.8*, and *PgrSIP1.2* showing a significant linear relationship with water accumulation in the juicy outer seed coat (Liu et al., 2021). Additionally, in *Lycium barbarum* fruit, most *LbAQP* genes were found to be down-regulated during ripening and showed a negative correlation with the relative water content of the fruit (He et al., 2022). Results from PCA (**Figure 7**) also indicated that genes in Cluster I, which are highly expressed in the pulp, were significantly positively correlated with pulp water content.

The efficiency of water transmembrane movement is influenced by both *AQP* expression levels and enzyme activity (Maurel et al., 2015; Kapilan et al., 2018; Rabeh et al., 2024). Several studies have established a connection between *AQP* gene expression and water loss in postharvest fruits. For instance, one study reported that in tomato fruit, the rate of water loss increased by 8.3% under 50 µM melatonin treatment, and the expression of *SlPIP12Q*, *SlPIPQ*, *SlPIP21Q*, and *SlPIP22* was up-regulated 2- to 3-fold (Sun et al., 2015). In pear fruit, the *PbPG* enzyme accelerated water loss by degrading cell wall polysaccharides, thereby expanding the intercellular layer and AQP-encoding gene expression (Ma et al., 2023). Additionally, repressing the expression of *CsPIP1;1*, *CsPIP2;4*, and *CsTIP1;2* alleviated water loss in citrus fruits (Zhang et al., 2021). The results of correlation analysis and PCA indicated that *AQPs* in Clusters I and III were positively correlated with average and cumulative weight loss, as well as water indexes such as water content, water potential, water potential gradient, and osmolytes in the pulp samples at 30, 60, 90, and 105 d along the PC1 direction (**Figure 7**). Notably, the pulp sample at 60 d formed a distinct cluster separate from the other pulp samples. This suggests that the *AQPs* that are abundantly expressed in the pulp, especially at 60 d, may facilitate efficient water transport from the pulp to the peel, thereby playing a crucial role in regulating fruit weight loss.

## 5 Conclusion

**Figure 8:**
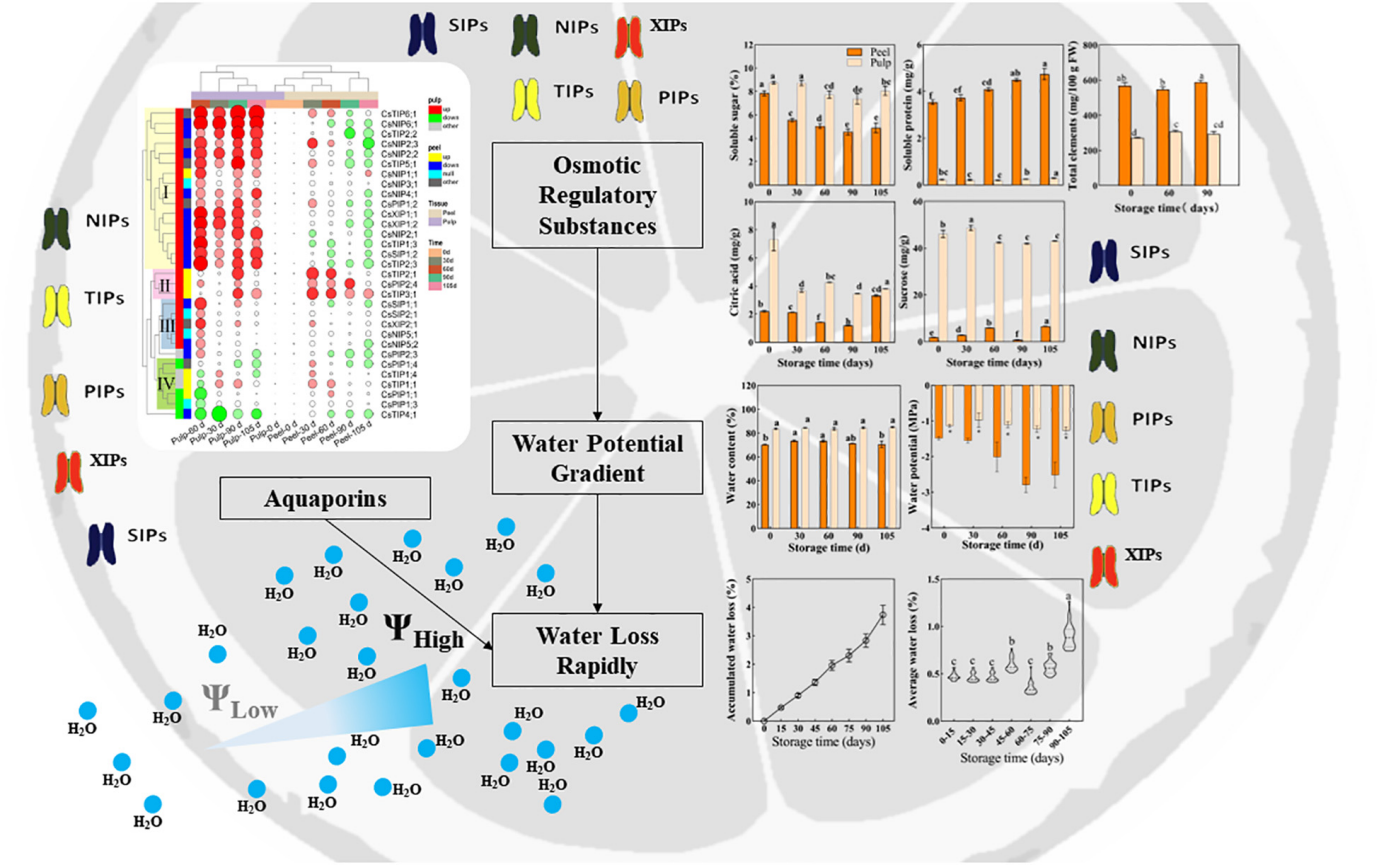
Schematic diagram of postharvest water loss of orange fruit.

Based on the aforementioned analysis, we can conclude that (as displayed in **Figure 8**) in Newhall navel orange fruit, water content and potential are higher in the pulp than in the peel and show minimal changes in the pulp during storage. Conversely, in the peel, water content increases before 60 d postharvest, and this is followed by a sharp decrease in the water potential after 60 d. This significant change in the water potential gradient between the peel and pulp after 60 d may contribute to an accelerated rate of water loss during later storage periods. Additionally, while osmolyte concentration decreases in the pulp, it increases in the peel over time. The expression pattern of *CsAQPs* exhibits a tissue-specific mode, with most genes being down-regulated in the peel and up-regulated in the pulp during storage. Notably, 16 specific *CsAQPs* are up-regulated in the pulp, particularly at 60 d postharvest, and are significantly positively correlated with weight loss, demonstrating their role in facilitating water transportation. Overall, these findings suggest that the differential regulation of dynamics between the pulp and peel, along with tissue-specific patterns of *CsAQP* gene expression, contribute to the modulation of water loss during the postharvest storage of Newhall navel orange fruit.

## Data Availability

The datasets presented in this study can be found in online repositories. The names of the repository/repositories and accession number(s) can be found in the article/**Supplementary Material**.
