# Supplementary material for: Osmolytes and CsAQP expression jointly influence water physiology in the peel and pulp of orange (Citrus sinensis (L.) Osbeck) fruit during postharvest water loss
Source: Front Plant Sci. 2024 Oct 21;15:1475574. doi: 10.3389/fpls.2024.1475574 (PMC11539049; doi:10.3389/fpls.2024.1475574)
Supplement: Supplementary file 1 [file Image1.pdf]

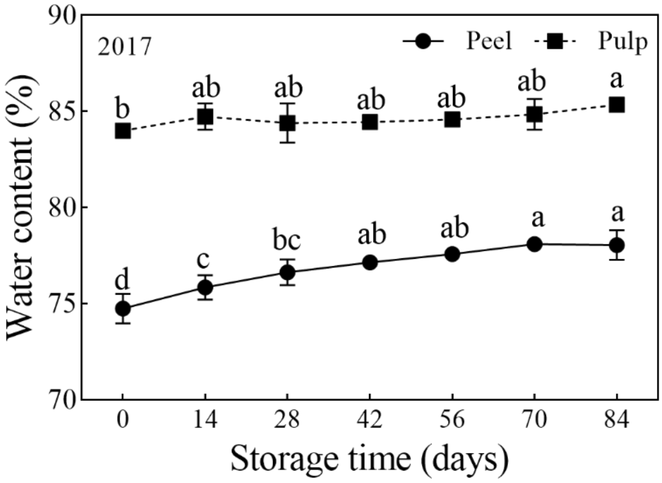

Supplemental Figure 1 Changes of water content in peel and pulp of Newhall navel orange during storage in year of 2017-2018. The values were presented as means ± SD (n ≥ 50). Different lowercase letters mean significant differences in the same tissue at different storage times with $P < 0.05$.

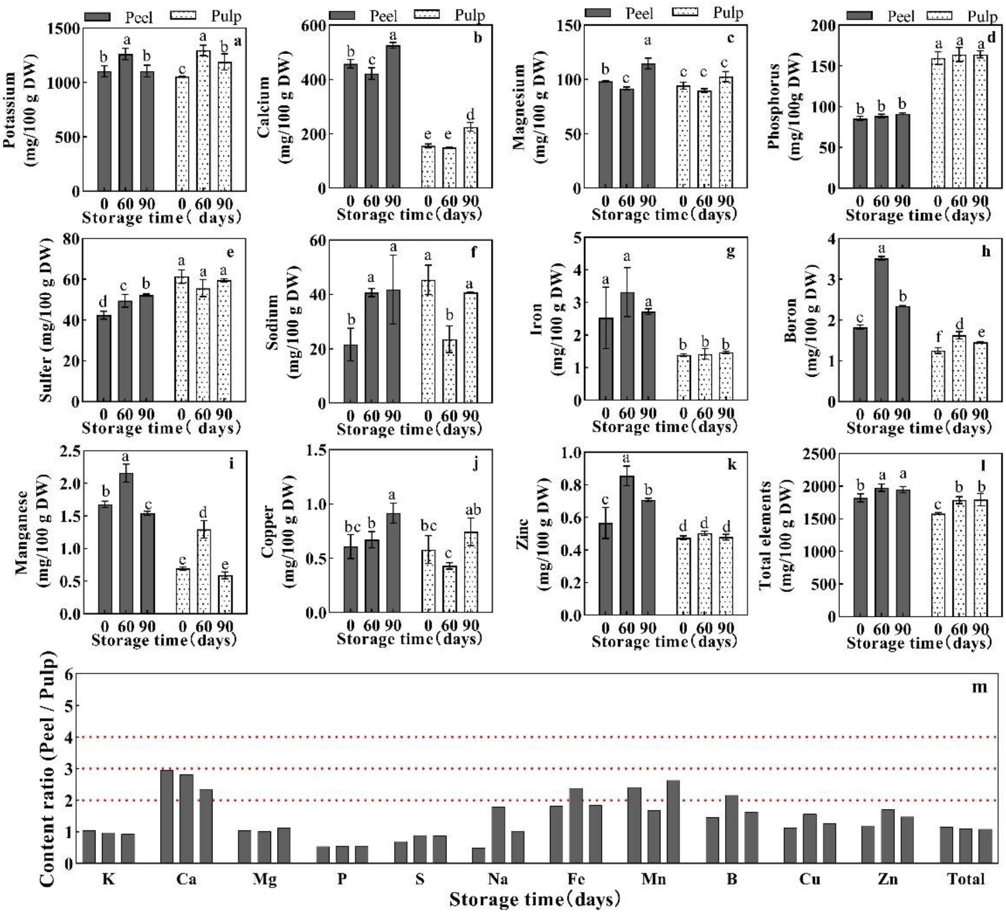

Supplemental Figure 2. Concentration of mineral elements in the peel and pulp of Newhall navel orange at 0 d, 60 d and 90 d post storage. (a) K, (b) Ca, (c) Mg, (d) P, (e) S and (f) Na, (g) Fe, (h) B, (i) Mn, (j) Cu, (k) Zn and (l) total element, and (m) the element concentration ratio of peel/pulp at the same storage period. The values were present as means ± SD (n=3). Different lowercase letters mean significant differences in the same tissue among different storage times with $P < 0.05$.
